# Neurodynamic mobilization and foam rolling improved delayed-onset muscle soreness in a healthy adult population: a randomized controlled clinical trial

**DOI:** 10.7717/peerj.3908

**Published:** 2017-10-13

**Authors:** Blanca Romero-Moraleda, Roy La Touche, Sergio Lerma-Lara, Raúl Ferrer-Peña, Víctor Paredes, Ana Belén Peinado, Daniel Muñoz-García

**Affiliations:** 1 Healthy Sciences Faculty, Camilo José Cela University, Madrid, Spain; 2 Departamento de Fisioterapia and Motion in Brains Research Group, Instituto de Neurociencias y Ciencias del Movimiento, Centro Superior de Estudios Universitarios La Salle, Universidad Autónoma de Madrid, Spain; 3 Laboratory of Exercise Physiology Research Group, Department of Health and Human Performance, School of Physical Activity and Sport Sciences-INEF, Technical University of Madrid, Madrid, Spain

**Keywords:** Muscle activation, Neurodynamic mobilization, Exercise-induced muscle damage, Recovery, Self-myofascial release, Pain

## Abstract

**Objectives:**

Compare the immediate effects of a Neurodynamic Mobilization (NM) treatment or foam roller (FR) treatment after DOMS.

**Design:**

Double blind randomised clinical trial.

**Setting:**

The participants performed 100 drop jumps (5 sets of 20 repetitions, separated by 2 min rests) from a 0.5-m high box in a University biomechanics laboratory to induce muscle soreness. The participants were randomly assigned in a counter-balanced fashion to either a FR or NM treatment group.

**Participants:**

Thirty-two healthy subjects (21 males and 11 females, mean age 22.6 ± 2.2 years) were randomly assigned into the NM group (*n* = 16) or the FR group (*n* = 16).

**Main Outcome Measures:**

The numeric pain rating scale (NPRS; 0–10), isometric leg strength with dynamometry, surface electromyography at maximum voluntary isometric contraction (MVIC) and muscle peak activation (MPA) upon landing after a test jump were measured at baseline, 48 h after baseline before treatment, and immediately after treatment.

**Results:**

Both groups showed significant reduction in NPRS scores after treatment (NM: 59%, *p* < .01; FR: 45%, *p* < .01), but no difference was found between them (*p* > .05). The percentage change improvement in the MVIC for the rectus femoris was the only significant difference between the groups (*p* < 0.05) at post-treatment. After treatment, only the FR group had a statistically significant improvement (*p* < 0.01) in strength compared to pre-treatment.

**Conclusion:**

Our results illustrate that both treatments are effective in reducing pain perception after DOMS whereas only FR application showed differences for the MVIC in the rectus femoris and strength.

## Introduction

Delayed-onset muscle soreness (DOMS) frequently occurs after exhaustive and/or unaccustomed exercise, particularly if the exercise involves eccentric muscle contractions. Eccentric contraction is characterized by high force generation and a low energy expenditure (Hedayatpour et al., 2008). Moreover, eccentric contraction often induces muscle fibre injury, which is associated with the muscle’s decreased ability to generate force and a set of indirect muscle damage markers, such as muscle soreness, decreased maximal voluntary muscle contractions, and increased muscle stiffness with reduced range of motion (ROM) (Kanda et al., 2013). The soreness that occurs during muscle fatigue typically arises the first day after the exercise and peaks in intensity by 48 h post-exercise (Torres et al., 2012).

This type of exercise causes a disruption of normal skeletal muscle banding patterns (alignment) and the broadening or complete disruption of sarcomere Z lines (Smith et al., 1994a). This leads to alterations in protein expression (Chen et al., 2003) and inflammation (Hubal et al., 2008), which play an important role in the muscle’s recovery and adaptation (Toumi & Best, 2003).

Various prophylactic and treatment strategies have been investigated in an effort to reduce the negative symptoms associated with unaccustomed eccentric exercise, including such strategies as nutritional supplementation, cryotherapy, electro-therapeutic modalities, and prior exercise (Ernst, 1998; Cheung, Hume & Maxwell, 2003; Connolly, Sayers & McHugh, 2003). These studies showed that anti-inflammatory drugs and massage reduced pain levels, but functional variables such as strength and ROM did not improve. A systematic review and meta-analysis by Torres et al. in 2012 demonstrated that therapeutic massage was the only intervention that positively affected function and the recovery from DOMS; however, the mean effect was too small to be considered clinically relevant. The evidence supporting the use of cryotherapy, as well as stretching and low-intensity exercise, is inconclusive (Torres et al., 2012). Currently, the foam-rolling massage is often used by athletes from many sports. However, there are only a few studies on the effects of foam-rolling massage and they have conflicting results regarding the improvements in ROM and muscular performance (MacDonald et al., 2013; Macdonald et al., 2014; Pearcey et al., 2015). In the same line, neurodynamic mobilization (NM) is a manual therapy method used to assess and treat neuromuscular disorders. It includes gliding techniques and tensile techniques. Gliding techniques or “sliders” are intended to produce a sliding movement between neural structures and adjacent nonneural tissues. NM has been shown to reduce pain and improve ROM (De-la Llave-Rincon et al., 2012). However, no studies have investigated its effects after exercise-induced muscle soreness or DOMS. Due to this, the purpose of this study was to assess the acute effects of a single NM treatment session on DOMS and to compare them with those of one foam roller (FR) session.

## Materials & Methods

Following the damaging plyometric exercise bout, the participants were randomly assigned in a counter-balanced fashion to either a FR or NM treatment using a computer-generated random-sequence table with a two-balanced block design (GraphPad Software, Inc, San Diego, CA, USA); treatments were administered 48-h post-exercise. The dependent variables were recorded before the exercise, 48-h post-exercise before treatment, and immediately post-treatment. The trial was registered with the United States National Institutes of Health Clinical Trials Registry, with the registration number NCT03160937.

### Subjects

**Figure 1:**
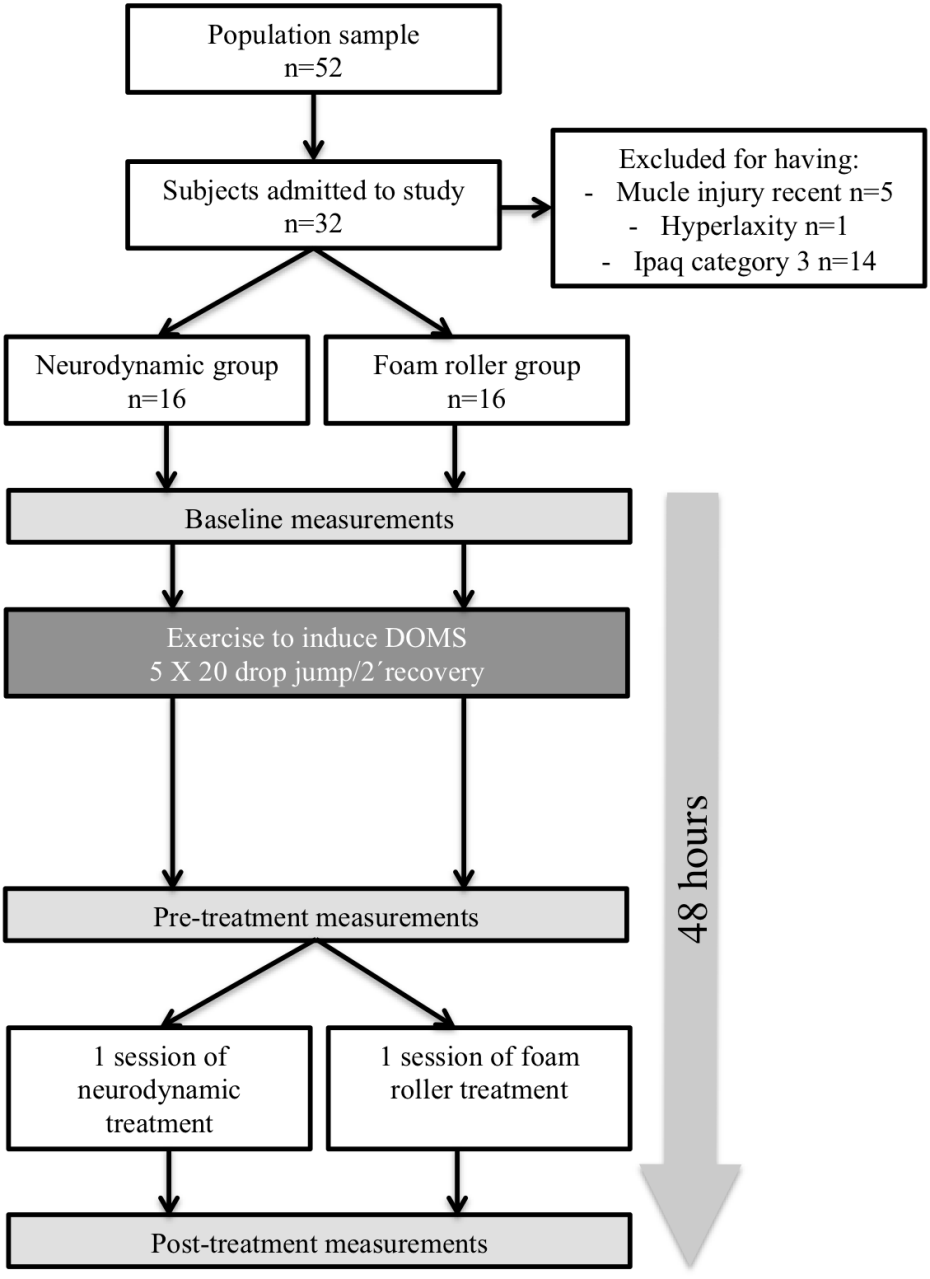
Flow chart of the study design.

Written informed consent was obtained from all subjects; moreover, the study design was described in detail to the subjects. All experimental procedures were ratified by the La Salle University Ethics Committee in accordance with the Helsinki Declaration (CSEULS-PI-009/2013). Thirty-two healthy subjects (21 male, 11 female; mean age =22.6 ± 2.2 years) participated in the study. All subjects were assessed by the International Physical Activity Questionnaire (IPAQ) questionnaire (Craig et al., 2003) and classified as moderately active (Category 2 in IPAQ). None of the subjects had a recent history of intensive training, heavy eccentric resistance, or plyometric exercise, and all subjects were free from musculoskeletal disorders in the last year. All subjects were asked to refrain from unaccustomed exercise during the experimental period, and the subjects abstained from all medications and dietary supplements during the experimental period and between testing sessions (Fig. 1)

### Induction of muscle soreness

Muscle soreness was induced with drop jumps. The required technique was demonstrated to all subjects before beginning the damaging bout, and they were coached during the protocol to be sure adequate technique and maximal effort in each jump was maintained. The participants performed 100 drop jumps (5 sets of 20 repetitions, separated by 2 min rests) from a 0.5-m high box. Upon dropping and landing, the subjects jumped vertically with maximal effort, landing on the same surface from which they had jumped. All participants performed 5 min on a static cycle to warm up before performing the plyometric exercise.

### Treatments

#### Foam roller group

**Figure 2:**
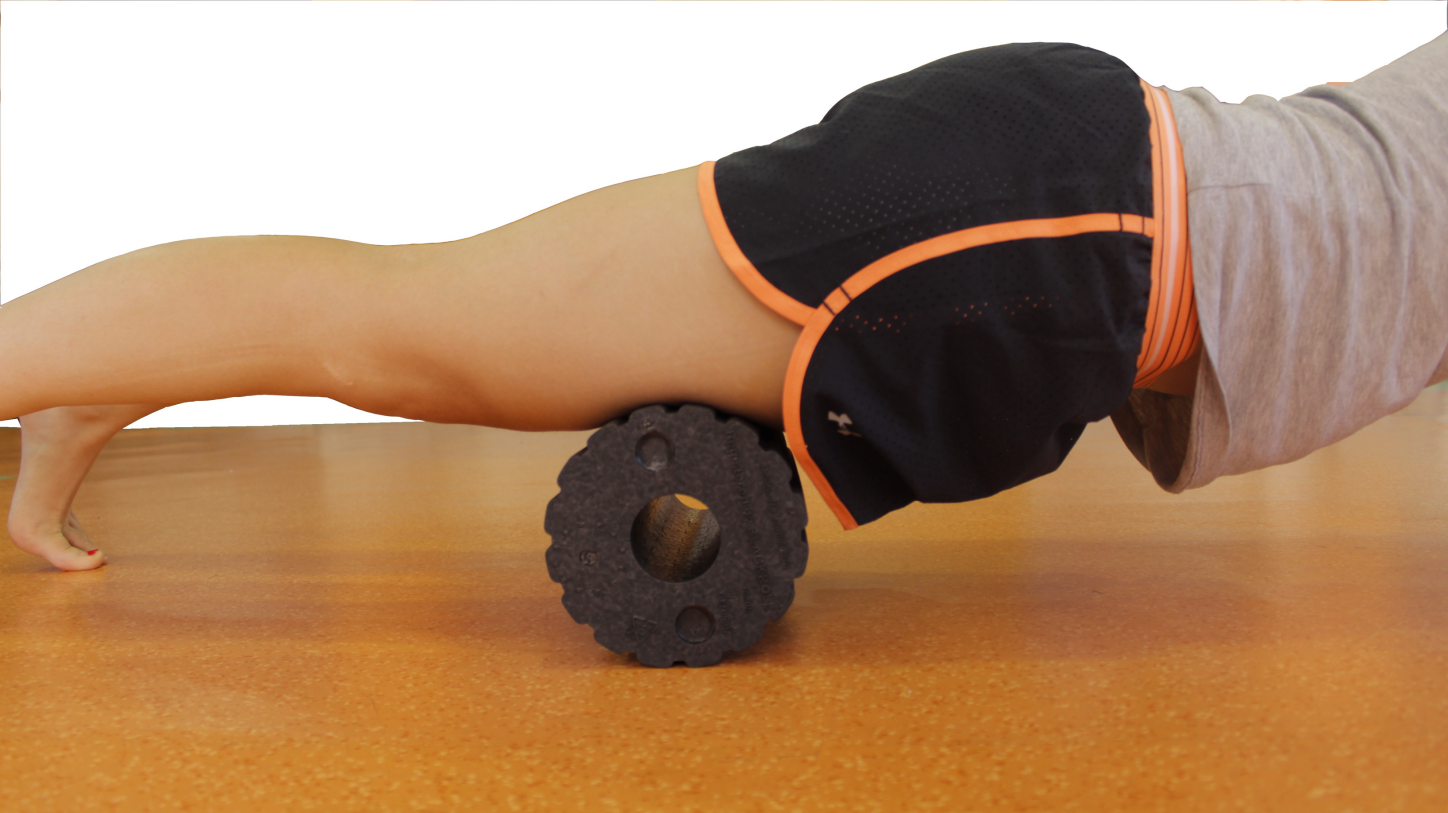
Myofascial foam-rolling technique.

The subjects included in the FR group performed the treatment using a custom-made foam roller composed of a uniform polystyrene foam cylinder (15-cm diameter × 90-cm long). The myofascial foam-rolling technique was based on a previously published protocol (MacDonald et al., 2013). The subjects began in the plank position with the foam roller at the most proximal portion of the quadriceps of both legs, with as much of their body mass as possible on the foam roller. They then rolled the foam roller down their quadriceps using short kneading-like motions until it was just above their patellae, and then rolled it back to its initial position in one fluid motion. The subjects repeated this motion for 1 min, rested for 30 s, and then repeated it again for 5 sets (Fig. 2).

**Figure 3:**
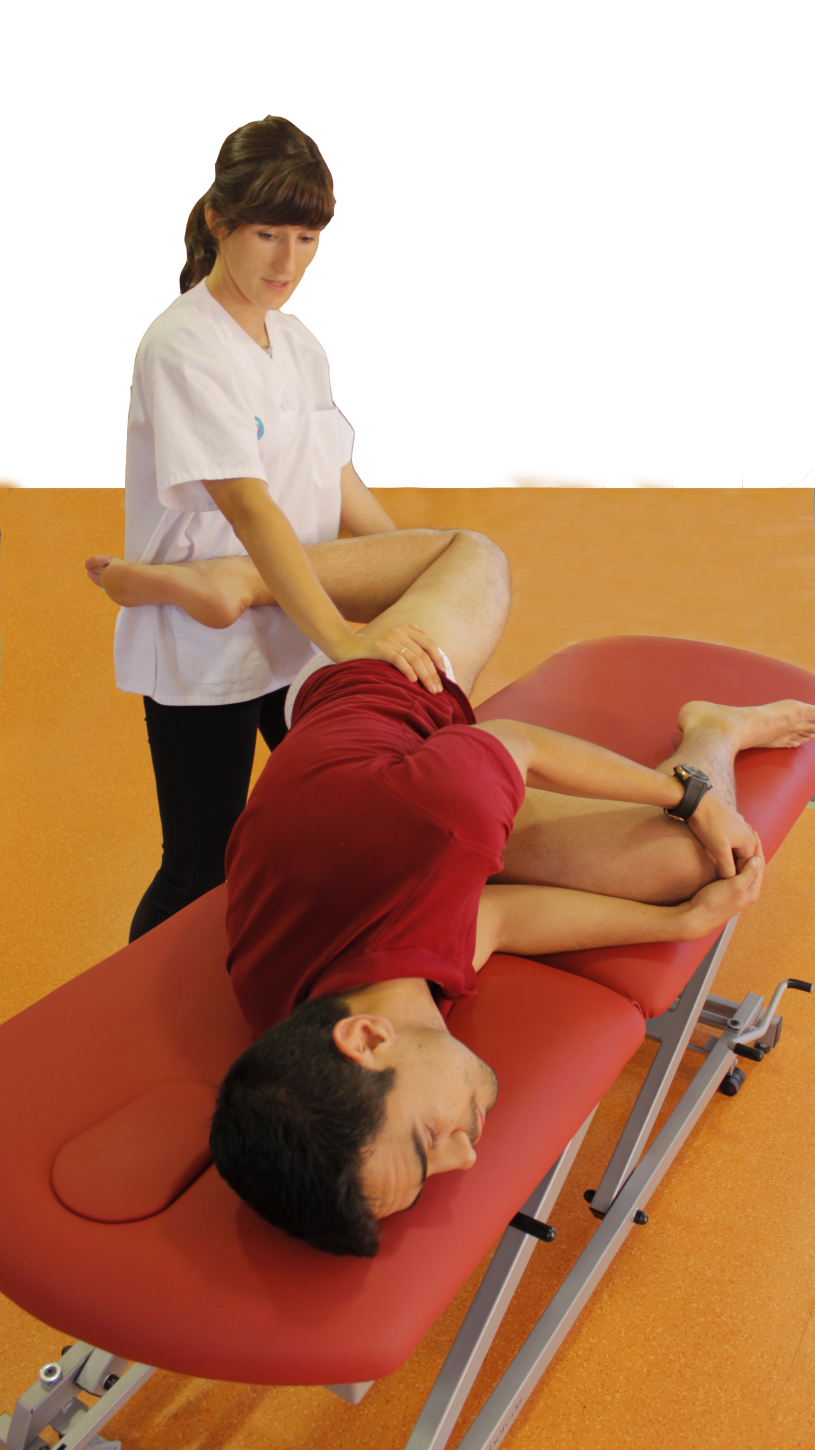
Femoral neurodynamic mobilization.

#### Femoral neurodynamic mobilization group

All participants were naive to the concepts of the NM technique. The protocol for the slump knee-bend NM technique was adapted from Butler (2000):

1. The participant was positioned lying on his/her side with a pillow slightly “cuddling” the underside leg (without fully flexing it) and the cervical and thoracic spines flexed. Standing behind the subject, the investigator supported the upper leg to maintain a neutral hip position (no abduction/adduction).
2. The knee of the upper leg was flexed and the hip extended (Fig. 3), either to the point of evoked or associated symptoms (P1), or until the resting symptoms began to increase (submaximal pain). If this point was not reached, the hip extension and knee flexion were stopped at the onset of firm resistance (R1).
3. When the symptoms were evoked, the subject was asked to extend his/her neck to achieve reduced neural tension of the neural tube. The mechanics of nerve sliding is essential for achieving a pain free technic while the investigator monitors for changes in the symptoms and resistance of the hip movement before ending the test.

The technique was repeated on both sides for 1 min for a total of 5 sets, with a 30-s rest between sets. The cadence for both techniques (FR and NM) was fixed at 3:4 using a metronome (free Iphone app; Gismar^®^).

### Primary outcome

The primary outcome was the change in the numeric pain-rating scale (NPRS; 0 = no pain; 10 = worst pain) used to measure the DOMS and to estimate the intensity of its associated pain (Farrar et al., 2001).

### Secondary outcomes

#### Surface electromyography (sEMG)

A Biosignalsplux pro^®^ (Plux Wireless Biosignals SA, Lisbon, Portugal) device was used to record muscle activity during the exercise protocol. Non-reusable circular Ag/AgCl electrodes with self-adhesive silicon were placed in a bipolar configuration with an inter-electrode distance of ∼20 mm (De Luca, 1997). The electrodes were connected by cable to a wireless analogue/digital signal converter with a resolution of 16 bits (Biosignalsplux pro^®^). The differential mode signal was detected with an input impedance of 100 G and 100 CMRR (common-mode rejection ratio) using a fifth-order Butterworth filter (10–300 Hz, 40 dB/dec). The electromyographic signal was amplified (overall gain = 1,000), captured at a sampling frequency of 1,000 Hz, properly identified, and then stored in a computer file for further off-line processing. The sEMG signal was managed using the European Surface EMG for Non-invasive Assessment of Muscles (SENIAM) guidelines (Hermens et al., 2000) criteria for signal normalization. Signal was filtered using a Band-pass filter previous to signal rectification. The root mean square was calculated in fixed time windows (0,05 seg) using the rectified signal. To ensure the best electromyographic signal quality, the skin was shaved and cleansed with alcohol before attaching the electrodes. The surface-EMG activity of the dominant leg, which was determined by asking the subject to kick a ball or step up onto a chair, was recorded via electrodes placed on the vastus medialis, vastus lateralis, and rectus femoris muscles as described by the SENIAM.

When measuring the maximal voluntary isometric contraction (MVIC) of the quadriceps, the starting posture was with 15° knee flexion (Lee et al., 2012). All subjects practiced the maximal isometric knee extension for the quadriceps in an isotonic knee-extension machine after two familiarisation trials (Vertical seated knee extension; Technogym, Gambettola, Italy) before the sEMG data was acquired. Each isometric trial lasted 5 s. Two measurements were obtained with a 2-min rest period between the repetitions, and the trial with the highest signal was calculated. The subject received consistent verbal encouragement during the MVIC. The maximal peak activation (MPA) was measured in two plyometric exercise repetitions (one drop jump from a 0.5-m high box) with a 2-min rest between repetitions.

#### Leg dynamometer measurements

The strength of the leg muscles was estimated with a Tecsymp Tkk5002 leg dynamometer (Tecsymp, Barcelona, Spain). The subject stood on the platform with the feet on marked tracks and the height of the handle was adjusted to keep the knees bent 120° while the trunk remained vertical. When a button was pressed, the subject performed an isometric leg extension, making the greatest possible effort. The attempt was not valid if the subject extended his/her back or pulled up the handle arms (Segovia, López-Silvarrey & Legido Arce, 2008). Three measurements were made with a 1-min rest between repetitions and the average was analysed after two familiarisation trials.

### Reliability of the measurements

One single evaluator who was blinded to the group assignments performed all tests that occurred 5 min prior to and 5 min after the intervention. All tests were performed in the same order before and after the intervention for all subjects to avoid any order effects.

With the exception of the sEMG, the means were calculated for the 3 measurements obtained for each variable by a blinded investigator. The measurement reliability and precision were quantified by calculating the intra-class correlation coefficient (ICC) with a 95% confidence interval using data acquired from five volunteers during two pre-exercise sessions using the leg dynamometer.

### Statistical analyses

The NPRS was chosen as the primary outcome measure. The magnitude of the effect was classified with the Cohen’s *d* coefficient as small (0.20–0.49), medium (0.50–0.79), or large (≥0.8) and was estimated to be small (effect size =0.29) (Cohen, 1988). To obtain a power of 0.90 and a *p*-level of 0.05, G*power software was used to estimate that 14 participants would be required for each group. All data analyses were performed with the Statistical Package for Social Sciences software, version 21.0 for Windows (SPSS Inc., Chicago, IL, USA). Descriptive statistics included the means and standard deviations. *P*-values less than 0.05 were considered statistically significant. A Shapiro–Wilk test was used to test the variables for normality (*p* > 0.05). With the exception of the NPRS and percentage change in strength, the data were not normally distributed. The percentage change was calculated with the standard formula: percentage change = [(pos *t*-test score − pre-test score)/pre-test score] × 100. Non-parametric statistics were used for the NPRS (absolute values), MVIC, MPA, and strength dynamometer results. The tests used to determine differences within groups were the Mann–Whitney *U* test, the Friedman test for analysing changes in the intragroup results, and the Wilcoxon signed-rank test for post-hoc intragroup comparisons. Intra-class correlation coefficients (ICC) and their 95% confidence intervals were utilized to determine the intra-tester reliability for the leg dynamometer measurements, which was good (ICC =0.90).

## Results

### Baseline characteristics

The baseline characteristics of the participants revealed no significant differences in age, height, weight, body mass index (kg/m^2^), or metabolic equivalent (MET) as estimated through the IPAQ questionnaire. The baseline values for the dependent variables did not differ between the groups (Table 1).

**Table 1:** Characteristics at baseline.

	NM group = 16			FR group = 16			
	Mean	SD	Range	Mean	SD	Range	*P*-value
Age	22.1	±4. 3	(18–32)	23.5	±4.8	(18–34)	0.41
Height (m)	1.75	±0.08		1.75	±0.09		0.69
Weight (kg)	68.18	±9.10		69.00	±12.02		0.10
BMI	22.12	±2.01		22.49	±2.28		0.67
METs	1272.09	±202.56		1303.38	±167.73		0.33

**Notes.**

Data are shown as mean ±SD.

NM Neurodynamic Mobilization group FR Foam Roller group BMI Body Mass Index MET Metabolic Equivalent of Task

**Figure 4:**
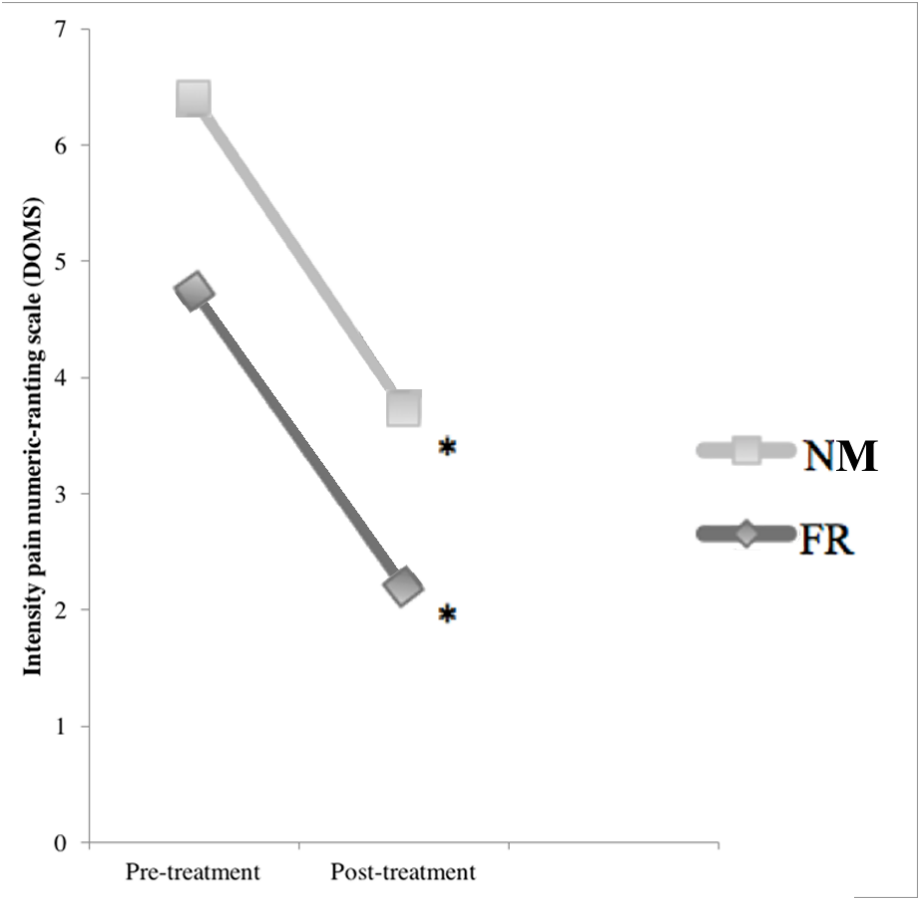
Comparison of the effects of DOMS and treatments: pre-treatment and post-treatment values in numeric pain rating scale. ^∗^ Significant differences in post treatment measure *p* ≤ 0.05.

None of the participants reported soreness during the baseline assessments before the intervention with plyometric exercise. The NPRS scores increased significantly 48 h after the exercise intervention (*p* < 0.01; Fig. 4). At that point, the median pain increment was 4.0 (3.0–6.7)for the NM group and 7.0 (4.3–8.0) for the FR group, with significant differences between the groups (*p* < 0.03). Both groups had significant reductions in their NPRS scores after treatment (NM: 2 (1.0–3.7), *p* < 0.01; FR: 3.5 (3.5–6.0), *p* < 0.01) which exceeds the minimal clinically important difference (Salaffi et al., 2004) (Table 2), with no significant difference between the groups (*p* > 0.05) in percentage change (Table 3).

### Neuromuscular performance of the quadriceps

After inducing the muscle soreness, the MVIC for the vastus medialis, vastus lateralis and rectus femoris muscles decreased significantly in both groups (*p* < 0.01) compared to baseline. After treatment, the vastus medialis and vastus lateralis improved significantly in both groups (*p* < 0.01); while the rectus femoris only significantly improved in the FR group (*p* < 0.01) compared to pre-treatment. The percentage change improvement in the MVIC for the rectus femoris was the only significant difference between the groups (*p* < 0.05) at post-treatment

The MPA was measured during drop jump. There were significant intra-group differences in the absolute values between peak activation at baseline, pre-treatment, and post-treatment for both groups (*p* < 0.01), but there were no significant differences between the groups (Table 4).

**Table 2:** Median and inter-quartil data of pain and strength.

		Baseline	Pretreatment	Posttreatment	Friedman (*p*-value)
Variable	Groups				
NPRS (0–10)	FR	0	7 (4.37–8.00)	3.5 (3.5–6.00)	0.01
	NM	0	4 (3–6.75)	2 (1–3.75)	0.01
Strength (Kg)	FR	135.15 (115.025–158.57)	122.30 (110.50–151.15)	131.25 (112.30–175.02)	0.02
	NM	138.35 (105.90–158.85)	129.35 (105.90–158.77)	141.15 (109.55–150.77)	0.17

**Notes.**

NPRS Numeric Pain Rating Scale Strength isometric hand held dynamometer FR Foam Roller group NM Neurodynamic Mobilization group

**Table 3:** Comparison in percentage change and effect size after treatment.

	FR group (*N* = 16)		NM group (*N* = 16)				
	Mean (%)	SD	Mean (%)	SD	Mean difference	95% CI	Effect size
NPRS	45.34	±22.92	58.61	±21.19	13.27	29.21 to −2.66	−0.29
Strength	8.55	±10.12	7.61	±17.6	0.93	11.13 to −9.43	0.03

**Notes.**

NPRS Numeric Pain Rating Scale Strength isometric hand held dynamometer FR Foam Roller group NM Neurodynamic Mobilization group

**Table 4:** Comparison in percentage change and effect size for EMGs activation.

Nonparametric tests of outcome data						
Change (%) Median (interquartile range)						
Variables group	MPA Vastus medialis	MPA Vastus lateralis	MPA Rectus femoris	MVIC Vastus medialis	MVIC Vastus lateralis	MVIC Rectus femoris
FR	9.09 (4.54–10)	4.58 (3.87–15.49)	11.27 (7.88–13.64)	8.09 (4.17–8.09)	5.13 (3.06–13.84)	7.04 (4.14–13.35)
NM	10 (7.28–10.34)	7.11 (0.42–10.94)	6.73 (2.5–6.73)	6.22 (4.04–9.17)	5.26 (0.73–10.33)	4.58 (0.00–9.58)
*U* Mann–Whitney	0.406	0.970	0.213	0.439	0.664	0.048

**Notes.**

MPA Maximal Peak Activation MVIC Maximal Voluntary Isometric Contraction FR Foam Roller group NM Neurodynamic Mobilization group

### Leg strength dynamometer measurements

**Figure 5:**
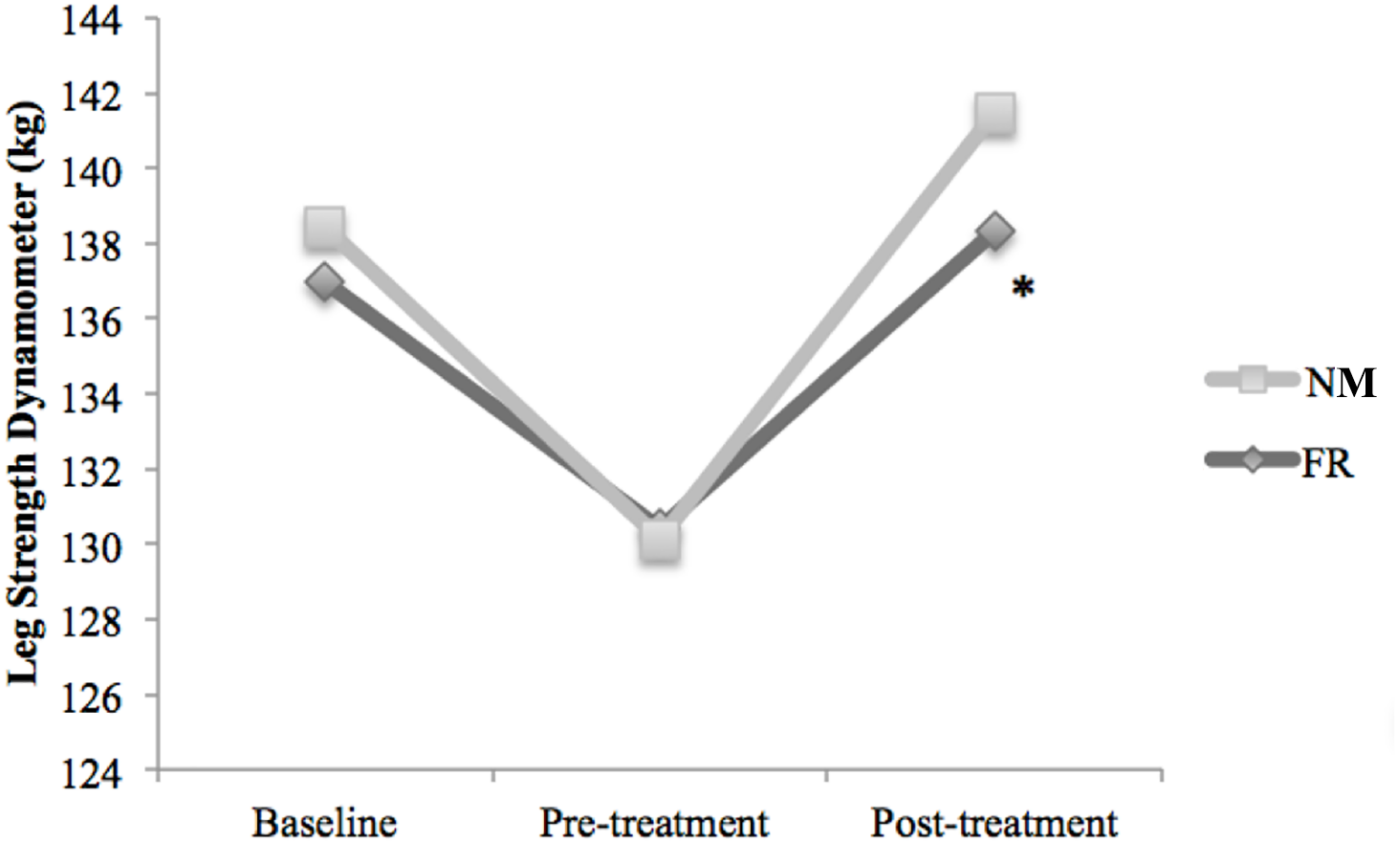
Comparison of the effects of DOMS and treatments: baseline, pre-treatment and post-treatment values in Kg for leg strength dynamometer. ^∗^ Significant difference in post treatment measure for the Foam Roller group *p* ≤ 0.05.

Figure 5 shows the leg strength values estimated with the dynamometer. Forty-eight hours after inducing the muscle soreness, leg strength had significantly decreased in the both groups (FR: −8.29%, *p* < 0.01; NM: −6.59%, *p* = 0.03). After treatment, only the FR group had a statistically significant improvement (*p* < 0.01) in strength compared to pre-treatment.

## Discussion

During high intensity training, DOMS may frequently appear and consequently decrease performance (Cheung, Hume & Maxwell, 2003). Currently, FR massage is used for recovery and to decrease pain perception (Schroeder & Best, 2015). On the other hand, NM is a manual method that has shown improvements for pain and ROM and may be another tool for athletes suffering from DOMS (De-la Llave-Rincon et al., 2012). This study examined the effects of a session of NM and FR after a bout of eccentric exercise that induced DOMS. To our knowledge, this is the first study that compared the impact of these interventions on DOMS and MVIC.

The main finding of this study was that both the FR and NM protocols were effective in reducing self-reported measures of DOMS. In agreement with our results in the FR group, many clinical trials have shown treatment effectiveness in soreness reduction (Beneciuk, Bishop & George, 2009; Lai et al., 2012; Castellote-Caballero et al., 2013; Healey et al., 2014; Jay et al., 2014). Clinical trials by Macdonald et al. (2014) and Pearcey et al. (2015) reported decreased soreness 24, 48, and 72 h after a foam-rolling intervention which was performed following a squat protocol consisting of 10 sets ×10 repetitions squat protocol. When Healey et al. (2014) compared planking to foam rolling prior to exercise, the fatigue rating (0–10) and soreness significantly decreased after the FR treatment.

A reason to explain the decreased perception of pain resulting from FR and NM treatments application is that parasympathetic activity is activated, changing hormonal levels and reducing cortisol concentrations (Best et al., 2008). Lund et al. (2002) suggested another theory, in which the pressure on the muscles during massage reduces mechanical hyperalgesia, which leads to the activation of descending inhibitory pathways.

Different studies on NM have also reported immediate hypoalgesia in asymptomatic participants (Beneciuk, Bishop & George, 2009; Lai et al., 2012) and in patients with chronic carpal tunnel syndrome (De-la Llave-Rincon et al., 2012). The study by Beneciuk, Bishop & George (2009) demonstrated that NM had an immediate hypoalgesic effect on C fibre-mediated pain perception (temporal summation), suggesting a hypoalgesic mechanism for the NM mobilization technique. Like these studies, both of our participant groups had significant reductions in pain as measured by their NPRS scores (NM =2.53 ± 0.92; FR =2.69 ± 1.41), which improved by 54% and 42%, respectively. These data can be interpreted as a “much better” clinical outcome (Salaffi et al., 2004).

In contrast to other clinical trials, our study found significant within-group differences in the MVIC between pre and post-treatment for the FR group, but not for the NM group. Both groups displayed deficits (−8% to −12%) after DOMS, however, only the FR treatment significantly improved MVIC for the rectus femoris muscle (9.29 ± 2.02%). The MVIC deficits were similar to those reported in previous DOMS studies (Zainuddin et al., 2005; Macdonald et al., 2014). However, we found no studies reporting that MVIC increased after FR or NM treatment to compare with these results. It is possible that the differences observed are due to rectus femoris being a biarticular muscle which may be impacted more so by the FR than the NM. In contrast, the NM treatment, hip extension is limited by neural limitations and so the rectus femoris is not specifically targeted. Some authors have reported good results in increasing range of motion, strength and muscle activation with the FR (Halperin et al., 2014; Macdonald et al., 2014; Pearcey et al., 2015). Pearcey et al. (2015) reported that FR treatment enhanced recovery from DOMS and reduced the observed decline in physical performance. Specifically, the FR treatment positively affected sprint speed, power, and dynamic-strength endurance at various time points after exercise in comparison with the control condition. The exact mechanism responsible for the improvement in performance remains unclear.

The current study showed an average decrease of 8% in leg strength as measured using dynamometry which subsequently improved by an average of 8% following FR. The decrease in isometric strength was similar as reported by previous literature (Robineau et al., 2012) in a half time soccer game simulation, which may explain injury risk (Marshall et al., 2014). Nevertheless, both treatments restored strength, and significant differences were not observed between them.

Other studies that have examined performance with vertical jump tests, have found no reductions in leg strength relative to baseline. This could be because of the important differences in FR application (five exercises targeting the major muscle groups) (Macdonald et al., 2014) or analysing participants with pain-free muscles (Halperin et al., 2014).

Decreases in muscle activity after exercise intervention could be due to factors such as a physiologic disruption of the structural proteins in the sarcomeres. Particularly, at the weakened Z lines as well as damage to the sarcolemma from the inhibition of cellular respiration and the accumulation of calcium that increases inflammation (Armstrong, 1984); along with, acute muscular fatigue that implies a decline in strength (Best et al., 2008); pain due to edema formation (Bobbert, Hollander & Huijing, 1986); or also from unusual patterns of muscle recruitment during movement (Nguyen et al., 2009; Sakamoto et al., 2009). Based on our MVIC measurements, the FR treatment promoted the recovery of muscle activity involving the rectus femoris, while the NM treatment did not.

Both the NM and FR treatments improved muscle pain and maximal peak activation measures. The most common reasons suggested for these improvements have been reduced edema, the enhanced removal of blood waste products, and improved tissue repair and healing (Weerapong, Hume & Kolt, 2005). A review by Cheung, Hume & Maxwell (2003) reported that increased blood flow enhances the removal of neutrophils and reduces prostaglandin production, thereby reducing any further damage associated with the inflammatory process (Crane et al., 2012).

Increased muscle activation and decreased pain may play an important role in preventing injuries, enhancing performance, and providing recovery treatment for pathologies that cause pain, especially in muscle and neural tissues. Also, the decrease in muscle soreness found in this study may enhance athlete compliance and could be easily incorporated into the daily training routine (Rutten et al., 2010).

A pilot study by Okamoto, Masuhara & Ikuta (2014) found other physiological effects in an investigation of the acute effects of FR treatment on arterial stiffness and vascular endothelial function. Measuring vascular activity and vascular stiffness through vasoactive substances such as nitric oxide (NO) and pulse wave velocity (PWV) measurements, respectively, this study showed that FR application had a beneficial influence on arterial function in healthy young adults. One FR treatment session decreased the PWV and increased the plasma NO concentration. This may be because mechanical stimuli such as arterial muscle compression induces arterial vasodilation, and while the magnitude is not affected by increased compression duration, it is enhanced by increasing the number of compressions (Tschakovsky & Sheriff, 2004). Compression might also distort the vascular endothelium, which could trigger the release of vasodilators such as NO. External leg compression causes elevated shear stress in the walls of the underlying vasculature by increasing blood-flow velocity in the deep veins of the extremities (Mayrovitz & Larsen, 1997), and shear stress on endothelial cells is a potent stimulus for NO production. Rapid cuff inflation might also increase shear stress on the vascular wall, likewise stimulating the endothelial release of NO (Liu et al., 1999). The subjects in the FR group repeatedly performed external compressions using the foam roller. These data suggest external compression that increases vasodilation might be a major pathway to increase the release of NO. The constant pressure on muscles during a FR treatment has also been shown to provoke biochemical changes reflecting less cellular stress and inflammation from increased neutrophil concentrations, increases in plasma creatine kinase (Smith et al., 1994b), and activated mechano-sensory sensors that indicate mitochondrial regeneration and stimulated muscle healing (Crane et al., 2012).

It should also be noted that the performance benefits and decreases in pain perception might be duration dependent. The studies reporting increased performance measures as a result of FR protocols utilized a minimum of 90-s FR applications (three 30-s sets [16] or two 1-min sets [28]), which is similar to our five 1-min sets, while one of the studies utilized a <30-s FR application (Healey et al., 2014) and reported no change in the performance measures (MacDonald et al., 2013).

There are possible concerns regarding pain perception differences between groups at the pre-treatment. Although subjects were randomly allocated to treatment groups of intervention, maybe a bigger sample size would balance this issue. Therefore, the percentage change was analyzed for comparing differences between groups. Also, another limitation is related to probes placement reliability. There are few options to avoid this limitation. The authors followed the SENIAM guidelines, but according to literature new procedures for sEMG measurements are required and new studies must be conducted for a standardization of the use of sEMG for research and for the use in the clinical settings. Finally, the short-term results of this study in participants with DOMS cannot be generalized to other population neither to the duration of the improvements obtained. Future research should compare the results with a placebo group as used elsewhere (Beneciuk, Bishop & George, 2009).

Further studies should focus on addressing other muscle groups and examining the effects of lengthening the FR treatment duration in repeated-measure designs.

## Conclusions

In conclusion, our results illustrate that both treatments are effective in reducing pain perception after DOMS whereas only FR application showed differences for the MVIC in the rectus femoris and strength. This important finding suggests they might be used to improve these variables in the general and athletic population.

##  Supplemental Information

10.7717/peerj.3908/supp-1 Supplemental Information 1 Raw data

10.7717/peerj.3908/supp-2 Supplemental Information 2 Consort

## Additional Information and Declarations

### Data Availability

The following information was supplied regarding data availability: The raw data has been supplied as Supplemental File.

### Clinical Trial Registration

The following information was supplied regarding Clinical Trial registration: NCT03160937.
